# Supplementary material for: Decentralized TB diagnostic testing with Truenat MTB Plus and MTB-RIF Dx vs. hub-and-spoke GeneXpert MTB/RIF Ultra in Mozambique and Tanzania: a cost and cost-effectiveness analysis
Source: PLOS Glob Public Health. 2025 May 30;5(5):e0004724. doi: 10.1371/journal.pgph.0004724 (PMC12124845; doi:10.1371/journal.pgph.0004724)
Supplement: S2 Text — Lists the members of the TB CAPT consortium and their affiliations. (DOCX) [file pgph.0004724.s003.docx]

TB-CAPT Consortium

Vinzeigh Leukes^1^, Adam Penn-Nicholson^1^, Morten Ruhwald^1^, Berra Erkosar^1^, Mikaela Watson^1^, Samuel G. Schumacher^1^, Sunita Singh^1^, Bernard Kivuma^2^, Muhuminu Nuru^2^, Francisca Chuwa^2^, Omary Ngome^2^, Neema Shija^2^, Deogratias Bulime^2^, Dorcas Mnzava^2^, Petro Sabuni^2^, Hosiana Temba^2^, Jamali Siru^2^, Jerry Hella^2^, Jonathan Msafiri^2^, Maja Weisser^2,16,23,24^, Mohamed Mbaruku^2^, Mohamed Sasamalo^2^, Alice Leonard^2^, Ambilikile Malango^2^, Annastazia Alexander^2^, Faith Komakoma^2^, Gloria Msigala^2^, Kasmir Johaness^2^, Grace Mhalu^2^, Robert Ndege^216,23^, Swalehe Masoud^2^, Theonestina Byakuzana^2,^, Mahmud Mahmud^2^, Lewis Batao^2^, Frederick Haraka^2^, Anange Lwilla^3^ Craysophy Zachariah^3^, Chacha Mangu^3^, Emmanuel Sichone^3^, Lonze Ndelwa^3^, Sara Kiula^3^, Alfred Danda^3^, Regino Mgaya^3^, Bariki Mtafya^3^, Issa Sabi^3^, Last Mwaipopo^3^, Nyanda Elias Ntinginya^3^, Raphael Edom^3^, Willyhelmina Olomi^3^, Delio Elisio^4^, Dinis Nguenha^4^,^22^, Edson Mambuque^4^, Joaquim Cossa^4^, Marta Cossa^4^, Neide Gomes^4^, Patricia Manjate^4^, Shilzia Munguambe^4^, Sozinho Acacio^4^, Belen Saavedra^4^, Helio Chiconela^4^, Katia Ribeiro^4^, António Machiana^5^, Bindiya Meggi^5^, Candido Azize Junior^5^, Carla Madeira^5^, Celso Khosa^5^, Claudio Bila^5^, Denise Floripes^5^, Ezequiel Nhamtumbo^5^, Jorge Ribeiro^5^ Sofia Viegas^5^, Albero Garcia-Basteiro^4,6^, Belén Saavedra^6^, Carole Amroune^6^, Joanna Ehrlich^6^, Laura de la Torre Pérez^6^, Sergi Sanz^6,25,26^, Friedrich Riess^7,20^, Katharina Kranzer^7,12,,20^, Michael Hoelscher^7,18,19,20^, Norbert Heinrich^7,18,19,20^, Sarah Mutuku^7,20^, Tejaswi Appalarowthu^7,20^, Leyla Larsson^7,20^, Maria del Mar Castro Noriega^8^, Claudia M. Denkinger^1,8,21^, Saima Bashir^8^, Daniela Maria Cirillo^9^, Elisa Tagliani^9^, Federico Di Marco^9^, Virginia Batignani^9^, Akash Malhotra^10, 29^, David Dowdy^10^, Claudia Schacht^11^, Julia Buech^11^, Caroline Stöhr^11^, Marguerite Massinga Loembé^13^, Pascale Ondoa^13^, Nqobile Ndlovu^13^, Fumbani Brown^13^, Yonas Ghebrekristos^14^, Cindy Hayes^14^, Ilse vanderwalt^14^, Shareef Abrahams^14^, Puleng Marokane^14^, Mbuti Radebe^14^, Neil Martinson^14^, Anura David^15^,Lesley Scott^15^, Pedro Da Silva^15^, Riffat Munir^15^, Wendy Stevens^14, 15^, Charles Abongomera^16,23^, Klaus Reither^16,23^, Leon Stieger^16,^ ^23^, Adrian Brink^17^, Chad Centner^17^, Helen Cox^17^, Judi van Heerden^17^, Mark Nicol^27^, Nchimunya Hapeela^17^, Parveen Brown^17^, Reyhana Solomon^17^, Widaad Zemanay^17^, Tania Dolby^28^.

^1^FIND, Geneva, Switzerland

^2^Ifakara Health Institute, Dar es Salaam, Tanzania

^3^Mbeya Medical Research Centre, National Institute for Medical Research (NIMR), Mbeya, Tanzania

^4^Centro de Investigação em Saúde de Manhiça (CISM) Manhica, Mozambique

^5^Instituto Nacional de Saúde (INS), Marracuene, Mozambique

^6^ISGlobal, Hospital Clínic – Universitat de Barcelona, Barcelona, Spain.

^7^Institute of Infectious Diseases and Tropical Medicine, LMU University Hospital, LMU Munich, Germany

^8^Department of Infectious Disease and Tropical Medicine, Heidelberg University Hospital, Heidelberg, Germany

^9^Emerging Bacterial Pathogens Unit, IRCCS San Raffaele Scientific Institute, Milan, Italy

^10^Johns Hopkins University Bloomberg School of Public Health, Baltimore, Maryland, USA

^11^LINQ Management GmbH, Berlin, Germany

^12^Clinical Research Department, London School of Hygiene and Tropical Medicine, London, UK.

^13^African Society for Laboratory Medicine, Addis Ababa, Ethiopia

^14^National Health Laboratory Service, South Africa

^15^WITS Diagnostic Innovation Hub, Faculty of Health sciences, University of the Witwatersrand, Johannesburg, South Africa

^16^Swiss Tropical and Public Health Institute, Allschwil, Switzerland

^17^Division of Medical Microbiology, University of Cape Town, South Africa

^18^Fraunhofer Institute for Translational Medicine and Pharmacology ITMP; Immunology, Infection and Pandemic Research, Munich, Germany

^19^Unit Global Health, Helmholtz Zentrum München, German Research Center for Environmental Health (HMGU), Neuherberg, Germany

^20^German Center for Infection Research (DZIF), Munich Partner Site, Munich, Germany

^21^German Center for Infection Research (DZIF), Heidelberg Partner Site, Heidelberg, Germany

^22^Department of Global Health and Amsterdam Institute for Global Health and Development, Amsterdam University Medical Centers Location University of Amsterdam, Amsterdam, Netherlands

^23^University of Basel, Basel, Switzerland

^24^Division of Infectious Diseases, University Hospital Basel, Basel, Switzerland

^25^Department of Basic Clinical Practice, Faculty of Medicine, University of Barcelona, 08007 Barcelona, Spain.

^26^CIBER Epidemiología y Salud Pública (CIBERESP), Instituto de Salud Carlos III, 28029 Madrid, Spain

^27^University of Western Australia, Australia

^28^National Health Laboratory Service, South Africa

^29^University of Washington, Seattle, USA
