# Supplementary material for: Decentralized TB diagnostic testing with Truenat MTB Plus and MTB-RIF Dx vs. hub-and-spoke GeneXpert MTB/RIF Ultra in Mozambique and Tanzania: a cost and cost-effectiveness analysis
Source: PLOS Glob Public Health. 2025 May 30;5(5):e0004724. doi: 10.1371/journal.pgph.0004724 (PMC12124845; doi:10.1371/journal.pgph.0004724)
Supplement: Attachment — Submitted filename: Response to reviewers.docx [file pgph.0004724.s005.docx]

The Editor

PLOS Global Public Health

We sincerely appreciate the time and effort that you and the reviewers have dedicated to evaluating our manuscript. The insightful feedback has helped us strengthen the clarity and robustness of our work, and we are grateful for the opportunity to address the comments provided. We have carefully responded to all reviewer comments. The responses can be found below this letter.

We appreciate your consideration and look forward to addressing any further outstanding comments. Thank you again for your time and for guiding this manuscript through the review process.

Kind regards,

Akash Malhotra, MPH

Corresponding author
Department of Global Health, University of Washington
Hans Rosling Center
3980 15th Ave NE, Seattle, WA 98105
amalhot7@uw.edu
+1 410 528 3747

Minor changes to the manuscript

1. We have added Dinis Nguenha as a co-author on this manuscript. They had contributed with trial implementation, cost data collection and had reviewed the manuscript, and their name was left off in the previous version owing to a minor oversight. Thank you for your kind understanding.
2. All three images have been converted to tif files, and satisfy the PACE recommendations.
3. We fixed a couple of minor spelling errors.

Please see edits based on the editor’s and reviewer’s comments below.

PGPH-D-24-02678
Decentralized TB diagnostic testing with Truenat MTB Plus and MTB-RIF Dx vs. hub-and-spoke GeneXpert MTB/RIF Ultra in Mozambique and Tanzania: a cost and cost-effectiveness analysis
PLOS Global Public Health

Dear Akash Malhotra

Thank you for submitting your manuscript to PLOS Global Public Health. After careful consideration, we feel that it has merit but does not fully meet PLOS Global Public Health’s publication criteria as it currently stands. Therefore, we invite you to submit a revised version of the manuscript that addresses the points raised during the review process.

Please submit your revised manuscript by Apr 06 2025 11:59PM. If you will need more time than this to complete your revisions, please reply to this message or contact the journal office at [globalpubhealth@plos.org](mailto:globalpubhealth@plos.org). When you're ready to submit your revision, log on to [https://www.editorialmanager.com/pgph/](https://urldefense.com/v3/__https:/www.editorialmanager.com/pgph/__;!!K-Hz7m0Vt54!jnTdFgA-EdV2Y3Czk5UKTDjpGzK0ppNCT4TAok2652lRjA5BpE1R_A2-dk9gEaHtt6Yfn8HJIa3FM1FnDaPHLQvLCI8$) and select the 'Submissions Needing Revision' folder to locate your manuscript file.

Please include the following items when submitting your revised manuscript:

- A rebuttal letter that responds to each point raised by the editor and reviewer(s). You should upload this letter as a separate file labeled 'Response to Reviewers'.
- A marked-up copy of your manuscript that highlights changes made to the original version. You should upload this as a separate file labeled 'Revised Manuscript with Track Changes'.
- An unmarked version of your revised paper without tracked changes. You should upload this as a separate file labeled 'Manuscript'.

Guidelines for resubmitting your figure files are available below the reviewer comments at the end of this letter.

We look forward to receiving your revised manuscript.

Kind regards,

Sizulu Moyo, MBCBH, MPH, PhD
Academic Editor
PLOS Global Public Health

Journal Requirements:

1. Please include a complete copy of PLOS’ questionnaire on inclusivity in global research in your revised manuscript. Our policy for research in this area aims to improve transparency in the reporting of research performed outside of researchers’ own country or community. The policy applies to researchers who have travelled to a different country to conduct research, research with Indigenous populations or their lands, and research on cultural artefacts. The questionnaire can also be requested at the journal’s discretion for any other submissions, even if these conditions are not met.  Please find more information on the policy and a link to download a blank copy of the questionnaire here: [https://journals.plos.org/globalpublichealth/s/best-practices-in-research-reporting](https://urldefense.com/v3/__https:/journals.plos.org/globalpublichealth/s/best-practices-in-research-reporting__;!!K-Hz7m0Vt54!jnTdFgA-EdV2Y3Czk5UKTDjpGzK0ppNCT4TAok2652lRjA5BpE1R_A2-dk9gEaHtt6Yfn8HJIa3FM1FnDaPHujfMkTE$). Please upload a completed version of your questionnaire as Supporting Information when you resubmit your manuscript.

Added sub-section in the Methods on Inclusivity in global research in lines 130 to 132. We have also attached the document as a supporting file.

Please review your reference list to ensure that it is complete and correct. If you have cited papers that have been retracted, please include the rationale for doing so in the manuscript text, or remove these references and replace them with relevant current references. Any changes to the reference list should be mentioned in the rebuttal letter that accompanies your revised manuscript. If you need to cite a retracted article, indicate the article’s retracted status in the References list and also include a citation and full reference for the retraction notice.

Thank you for raising this. We can confirm that the order of the references is up to date.


[Note: HTML markup is below. Please do not edit.]

Reviewers' comments:

Reviewer's Responses to Questions

**Comments to the Author**

1. Does this manuscript meet PLOS Global Public Health’s [publication criteria](https://urldefense.com/v3/__https:/track.editorialmanager.com/CL0/https:*2F*2Fjournals.plos.org*2Fglobalpublichealth*2Fs*2Fcriteria-for-publication/1/010f0195712220a5-16e0e99c-7812-4672-8018-ec0e82bb5c49-000000/rbJFgVaXf6ZzwFqslbh3_6fyFbAOiH6DqwanU8tkWXY=201__;JSUlJSU!!K-Hz7m0Vt54!jnTdFgA-EdV2Y3Czk5UKTDjpGzK0ppNCT4TAok2652lRjA5BpE1R_A2-dk9gEaHtt6Yfn8HJIa3FM1FnDaPH4UeiN4A$)? Is the manuscript technically sound, and do the data support the conclusions? The manuscript must describe methodologically and ethically rigorous research with conclusions that are appropriately drawn based on the data presented.

Reviewer #1: Yes

Reviewer #2: Yes

2. Has the statistical analysis been performed appropriately and rigorously?

Reviewer #1: Yes

Reviewer #2: Yes

3. Have the authors made all data underlying the findings in their manuscript fully available (please refer to the Data Availability Statement at the start of the manuscript PDF file)?

The [PLOS Data policy](https://urldefense.com/v3/__https:/track.editorialmanager.com/CL0/https:*2F*2Fjournals.plos.org*2Fglobalpublichealth*2Fs*2Fdata-availability/1/010f0195712220a5-16e0e99c-7812-4672-8018-ec0e82bb5c49-000000/yIWmpW4i8KPUYm3mx7NkVSCPZ1Q-AOUe5hGRJeNSC-c=201__;JSUlJSU!!K-Hz7m0Vt54!jnTdFgA-EdV2Y3Czk5UKTDjpGzK0ppNCT4TAok2652lRjA5BpE1R_A2-dk9gEaHtt6Yfn8HJIa3FM1FnDaPHUxs1DL4$) requires authors to make all data underlying the findings described in their manuscript fully available without restriction, with rare exception. The data should be provided as part of the manuscript or its supporting information, or deposited to a public repository. For example, in addition to summary statistics, the data points behind means, medians and variance measures should be available. If there are restrictions on publicly sharing data—e.g. participant privacy or use of data from a third party—those must be specified.

Reviewer #1: Yes

Reviewer #2: Yes

4. Is the manuscript presented in an intelligible fashion and written in standard English?

PLOS Global Public Health does not copyedit accepted manuscripts, so the language in submitted articles must be clear, correct, and unambiguous. Any typographical or grammatical errors should be corrected at revision, so please note any specific errors here.

Reviewer #1: Yes

Reviewer #2: Yes

5. Review Comments to the Author

Please use the space provided to explain your answers to the questions above. You may also include additional comments for the author, including concerns about dual publication, research ethics, or publication ethics. (Please upload your review as an attachment if it exceeds 20,000 characters)

Reviewer #1: Malhotra and colleagues have examined the costing and cost effectiveness of two TB testing strategies, a decentralised testing approach versus a hub and spoke approach. Both strategies use commercially available NAATs. This was a study nested within the TB CAPT trial, the settings of which were Mozambique and Tanzania. Decentralised TB testing was found to be more cost-effective than a hub and spoke approach. This is an interesting and well-presented manuscript, and the study seems to have been well-done. The findings are a valuable contribution to the TB costing literature, particularly for programs looking at implementing different TB testing strategies. I have some specific comments and questions to strengthen reporting, particularly regarding clarity of outcomes/analysis section.

Comments
Line 54-56: what were the results for 60 days post-enrolment? This is mentioned in the methods, but results aren’t included here so seems a bit incomplete.
Thank you for catching that. We have now added the following to lines 56 to 58: *“At sixty days, costs per treatment initiation were $581($493, $706) for hub-and-spoke vs. $678($576, $811) for decentralized testing in Mozambique, and $391($324, $476) vs. $591($494, $716) in Tanzania.”* And to lines 60-61 we have added: “*and $805(-$10107, $10560) and -$353(-$20299, $20802) for sixty-day treatment initiation, respectively.*”

Line 74-5: this is a critical rationale for conducting this work, but I am not sure if Reference 6 if the best study to cite here, since this is another costing/modelling study, rather than work that showed that centralised testing resulting in missed diagnoses and patients lost to follow-up. Suggest verifying if this is ideal citation to include and perhaps add another citation to strengthen claim.
We appreciate your valuable suggestion. We have now added the following citation to complement the existing citation in line 77-78: “*Cattamanchi A, Berger CA, Shete PB, et al. Implementation science to improve the quality of tuberculosis diagnostic services in Uganda. Journal of Clinical Tuberculosis and Other Mycobacterial Diseases 2020; 18: 100136.”* to better convey our rationale. The study explicitly discusses gaps in the cascade of care with referral based Xpert testing.

Line 93-95: I think it may be helpful to sharpen up terminology in this sentence. I assume that by ‘traditional model’, what is meant is ‘hub and spoke’ – if so, please modify in text. As well, by ‘decentralised’ here, does that mean bringing the tests to people who need testing (e.g., ACF kind of approach), as opposed to passive case finding? Or is it referring to test placement? It seems that simply placing a test in a de-centralised location (e.g., in rural areas) may still require multiple visits for obtaining a TB diagnosis as people may not be able to wait around for an hour+ – so perhaps considering adding a bit of detail to more fully explain what is meant by ‘decentralised’.

We thank you for this suggestion and have clarified the terminology in the text. By "traditional model," we are referring to the hub-and-spoke system, where testing occurs at higher-level health facilities (e.g., district/provincial/referral hospitals or centralized laboratories). By "decentralized testing," we mean the placement of Molbio Truenat machines in peripheral health facilities, allowing testing to occur closer to where people seek care. We have modified the text accordingly to ensure clarity. The sentence has now been tweaked to the following in lines 97 to 99 for clarity: “*Decentralized testing with Molbio Truenat at peripheral health facilities allows for accurate and rapid, same-visit results, contrasting with the traditional hub-and-spoke model at centralized hospitals or laboratories, thereby improving access and lowering out-of-pocket costs.”*

Line 106-111: I am not very familiar with TB CAPT (sorry) but wanted to confirm that the unit of randomisation was the clinic/peripheral health facilities? So each clinic included would have at baseline been doing hub-and spoke testing, but would have had the potential to start using TrueNat testing? Perhaps a couple of sentences about the eligibility criteria of the healthcare facilities could be included, to provide a bit more context for readers who aren’t familiar with Mozambique or Tanzania settings.

Thank you for your comment. We had initially omitted this information since it was published in our protocol paper. However, we agree that providing additional context will help readers. Clinics were indeed the unit of randomization, with all sites initially following the hub-and-spoke model. Facilities were eligible if they offered TB treatment, did not have an on-site GeneXpert laboratory, and were selected in coordination with the National TB Programs. Stratification was based on location and estimated TB notification volume.

We have now updated the manuscript in lines 109 to 113 with the following addition: *“The unit of randomization was the clinic. Clinics were eligible for inclusion if they offered TB treatment, did not have a GeneXpert laboratory on-site, and were selected in coordination with the respective National TB Programs. Selection was based on TB notification data from 2018–2019, and clinics were stratified by location and estimated TB testing volume before randomization.”*

Line 118-120: Some questions about the participant costs: Can you specify the number of people included as the basis for participant costs? Are you confident that these individuals are representative of the population in the sampling frame? How were the participant costs measured? Survey administered to participant? Survey completed by trial staff? Interview? Something else? Please specify in-text.
Thank you for your comment. In lines 204-206, under the Results, we specify the number of participants enrolled in the participant costs survey, and have the requisite detail in Table 1. The manuscript states: “*Of 3987 participants in the TB-CAPT Core trial, 388 (9.7%) were enrolled in the participant cost survey. Survey participants were similar to the full study population in most measured characteristics (Table 1). Survey respondents were more likely to be female in Mozambique.”*

In lines 135 to 137 of the methods we state that we obtained participant costs from approximately every 10^th^ participant enrolled and are confident that these are representative of our study population to a reasonable degree, as demonstrated in Table 1.

Based on your suggestion, we have now clarified in the Methods, in lines 137-138: *“**Participant costs were captured through a structured participant cost survey, with interviews directly administered to participants by study staff.”*

Line 130: Some questions about the health facilities selected for the health system costs estimates: how were the 19/29 clinics selected? Was this pre-specified? How many of each arm were included? Do you think these clinics are representative of all studied facilities?

Thank you for your comment. The 19/29 clinics included in the health system cost estimates were selected based on a combination of logistical feasibility and site managers’ judgment. Each country was requested to provide data from a minimum of 2 clinics in each arm (intervention and control), ensuring heterogeneity in clinics when looking at testing volume. In Tanzania, 6 intervention and 6 control clinics were included, while in Mozambique, 4 intervention and 3 control clinics contributed data. The specific clinics were not pre-specified by the analysis team. Instead, selection was based on the ability to administer surveys to clinic staff and practical considerations at the site level. While we cannot be certain that these clinics fully represent all study facilities, we believe the selection process, which incorporated site-level expertise, provides a reasonable degree of representativeness.

Outcomes: I’m having a bit of a hard time following this section and distinguishing between ideas. There are effectiveness and cost-effectiveness outcomes. Is ‘the facility-based diagnostic cost per participant tested’ and the ‘diagnostic cost per participant starting treatment within seven days of enrolment’ cost or effectiveness outcomes? Something else?
Thank you for your comment. the effectiveness outcomes will be presented in the main trial manuscript (which is currently under review at Lancet Primary Care). We introduce the outcome of the main trial in the first sentence in lines 163 to 164: “The primary effectiveness outcome in the parent TB-CAPT Core trial was the number of participants with microbiologically confirmed pulmonary TB initiating treatment within one week of enrolment.” **We have not conducted this analysis in our specific paper.**

To be clear, following up on your comment, we have slightly modified lines 164 to 166 to read: “*As cost outcomes, we estimated* *the facility-based diagnostic cost per participant tested, as well as the* *diagnostic cost per participant starting treatment within seven days and sixty days of enrolment*”.

Please note, in our economic evaluation we specifically analyze the following:

1. The facility-based diagnostic cost per participant tested. Here the **denominator is the number of people tested.** This is a cost outcome.
2. Diagnostic cost per participant starting treatment within seven days of enrolment. **Here the denominator is number of participants starting treatment within seven days and sixty days**. This is a cost-effectiveness outcome. We had missed mentioning sixty days in this paragraph and have made this correction in line 166.
3. We then compare the intervention arm to the standard of care to present the incremental facility-based diagnostic cost per incremental microbiologically positive participant initiating treatment within seven days of enrolment. This is the primary cost-effectiveness outcome of this trial. Here, **the denominator is the difference in the treatment initiations between the arms.**
4. We have also conducted the same analysis as in #3 for the sixty-day period, which is the secondary cost-effectiveness outcome.

Analysis: Writing is direct and not overly technical. However, after reading through this section a few times, I am still not sure what outcomes/calculations are being discussed in each paragraph:
Line 160: regarding the deterministic sensitivity analysis, ‘evaluate the influence of key assumptions’ on what specific outcome? Mean facility-based diagnostic cost per px tested? Something else? Please specify this in-text.
We apologize for the confusion. The deterministic sensitivity analysis is being performed on the primary cost-effectiveness outcome. Based on your helpful suggestion have now added the following to line 180-181: “facility-based diagnostic cost per incremental microbiologically positive participant initiating treatment within seven days of enrolment”.

Line 163-169: For the probabilistic sensitivity analysis, some explanation of assumption(s) being tested would be welcome – in other words, why were these sensitivity analyses done? I think it was to see the impact of varying each cost component, but I don’t quite follow why varying the ‘effectiveness’ value would be helpful (as isn’t effectiveness an outcome? [ie., the number of px with confirmed pulmonary TB starting treatment within 7 days]).
Thank you for your comment. The probabilistic sensitivity analysis was conducted to assess how variations in both **cost inputs and epidemiological effectiveness outcomes** impact the cost-effectiveness estimates. While “effectiveness” is an outcome, it is influenced by **underlying disease prevalence**, which in turn affects the number of people testing positive for TB. In settings with higher TB prevalence, more individuals would be diagnosed and initiated on treatment, potentially improving cost-effectiveness, whereas in lower-prevalence settings, fewer individuals would be diagnosed, impacting the incremental cost per treatment initiation. By varying effectiveness values within plausible ranges, we aimed to capture the **uncertainty in real-world implementation scenarios** and ensure robust cost-effectiveness estimates.

Based on your helpful suggestion, we have clarified this in lines 187 to 189: *“In addition to costs, effectiveness values were varied to account for differences in TB prevalence, which influence the number of individuals testing positive and subsequently impact cost-effectiveness estimates.”*

Line 170: how are testing volumes different from testing frequency? Consider indicating in-text
Outcomes + Analysis: perhaps the order of information presented in the Analysis sub-section could follow the order of information in the Outcomes section for improved clarity?
Thank you for your thoughtful comment. We acknowledge that testing volume and testing frequency have been used somewhat interchangeably. We used frequency to also indicate how operational constraints in a lab could impact the number of tests conducted. Essentially, volume is a product of frequency and the time period over which testing occurs.

Line 171: I think it may be pertinent to mention earlier than the Truenat machines were dual systems. Perhaps in “study design and participants” section this could be mentioned

Thank you for your helpful suggestion. We have added the following in lines 115-116: *“capable of processing two samples simultaneously”*

Reviewer #2: Peer Review comments: Cost-effectiveness of decentralized TB testing with Truenat vs. GeneXpert

Summary:
The study assessed the cost and cost-effectiveness of identifying additional TB patients using the Molbio Truenat platform with MTB Plus and MTB-RIF Dx assays (Truenat MTB assays), in comparison to the hub-and-spoke Xpert MTB/RIF Ultra system (standard of care). The study highlights the potential for Truenat MTB assays to deliver accurate and rapid, same-visit results for TB diagnosis. The analysis provides valuable insights into the cost-effectiveness of the Truenat MTB assays compared to the Xpert MTB/RIF Ultra system, showing that decentralized TB testing with Truenat MTB assays is cost-effective relative to hub-and-spoke testing Xpert MTB/RIF Ultra system, in Mozambique and Tanzania. However, certain aspects of the study design, particularly the population used, outcome measures, and implementation algorithm, could be clarified further for the reader to follow easily.

Population and Outcomes
It was not very clear to me how the study populations were used to define the outcomes. Specifically, why was the diagnostic outcomes are measured based on individuals enrolled in the study (i.e. line 50;). A clearer explanation of the outcome would be helpful.

Thank you for your comment. We assume you meant the Outcomes section in line 162, not line 50.

We measured diagnostic outcomes based on enrolled participants because they represent people actively seeking care for TB symptoms, making the findings more relevant for real-world implementation. Since TB diagnosis is influenced by when one gets tested and initiated to treatment, using the study population lets us assess diagnostic performance and cost-effectiveness under programmatic conditions while keeping comparisons across arms consistent.

• Per patient tested: The report uses this as a primary outcome measure; however, it would be beneficial to discuss the relevance of this metric in assessing cost-effectiveness from a clinical perspective.

Thank you for this suggestion. We agree that discussing the clinical relevance of cost per patient tested would add value. While this metric is widely used in economic evaluations, its direct clinical interpretation is limited. However, it remains important for understanding resource use and efficiency, particularly in settings where testing capacity and costs are major constraints. To address this, we have added the following to the Discussion section acknowledging the clinical implications of this metric and its role in cost-effectiveness assessments. Lines 290 to 293 state:

“Cost per patient tested provides a useful measure of resource use and efficiency, especially in settings where diagnostic capacity and budget constraints shape decision-making. In decentralized testing models, understanding the cost per test helps guide optimal placement of diagnostic platforms to balance accessibility, affordability, and impact.”

• Per patient initiated on treatment within 7-60 days of enrolment: Clarify in the methods why treatment initiation is considered within 7-60 days of enrolment rather than from the point of diagnosis? A clear justification for this choice would strengthen the methodology.

Thank you for your comment. We measured treatment initiation within 7-60 days from enrolment rather than from the point of diagnosis to account for potential delays in receiving a diagnostic test, which can vary across and within study arms due to clinic workflow, patient follow-up, and access barriers. This approach captures both diagnostic and treatment delays, providing a more comprehensive assessment of real-world implementation. We added the following in lines 93-94: *“accounting for potential delays in receiving a diagnostic test due to clinic workflow, patient follow-up, and access barriers”.*

• Implementation Algorithm: It would be useful to have a brief description of the implementation algorithm for the diagnostic tools used in both arms of the comparison. Specifically:
Thank you for your comment. Please see our responses below.

• Population tested: Is the population comprised of passive individuals or TB care-seeking individuals?
In lines 113-114 we mention “*Adults presenting with symptoms suggestive of tuberculosis were enrolled across the 29 health facilities”.*

• Testing algorithms: What are the specific steps or protocols followed for testing in each arm?
We have now indicated the following in lines 120 to 124 of the Methods: “At intervention clinics, microscopists were trained to use the Truenat platform, perform assays, and ensure rapid result communication for same-day treatment initiation, with repeat or reflex testing conducted as needed. At control clinics, staff received refresher training on national TB guidelines, with sputum samples processed via smear microscopy on-site or Xpert testing off-site, while operational bottlenecks like sample transport delays and stockouts were addressed through logistical provisions.”

• Clinical performance comparison: Are the Truenat MTB assays and the Xpert MTB/RIF Ultra system comparable in terms of clinical performance, specifically sensitivity and specificity? i.e. if the Truenat assays have lower specificity, it could result in unnecessary treatment being initiated, which should be carefully considered in the cost-effectiveness analysis.

Yes, these systems are comparable, as indicated in the study protocol and this paper by Penn-Nicholson et al., “A prospective multicentre diagnostic accuracy study for the Truenat tuberculosis assays”. doi: [10.1183/13993003.00526-2021](https://doi.org/10.1183/13993003.00526-2021)

Additional: Do you have results showing patient-level costs (alone) by the different study arm and were there any significant differences between the arms to show that the decentralized TB testing with Truenat MTB reduces patient costs?

Thank you for your question. We do have patient-level cost results by study arm; however, these findings will be presented in a separate extended cost-effectiveness analysis (ECEA) in a few months. To avoid duplication of results, we have not included a detailed breakdown in this manuscript.

Minor:
1. Abbreviations: Spell out some of the abbreviations: DALY, QALY, some diseases.

Thanks for catching this. We have now spelt out the abbreviations where they first appear throughout the manuscript.

2. Time Horizon for Costs: I may have missed this, but, the authors can also clarify the time horizon for which the reported estimated costs.

Thank you for your suggestion. We added the following to lines 160 and 161: “*The cost analysis used a time horizon corresponding to the trial period, capturing costs incurred during the study.”*

6. PLOS authors have the option to publish the peer review history of their article ([what does this mean?](https://urldefense.com/v3/__https:/track.editorialmanager.com/CL0/https:*2F*2Fjournals.plos.org*2Fglobalpublichealth*2Fs*2Feditorial-and-peer-review-process*23loc-peer-review-history/1/010f0195712220a5-16e0e99c-7812-4672-8018-ec0e82bb5c49-000000/-7yKekFOsoEXgJEu8CBgwg7nsR7MdVk-VKH-Z0vTtE0=201__;JSUlJSUl!!K-Hz7m0Vt54!jnTdFgA-EdV2Y3Czk5UKTDjpGzK0ppNCT4TAok2652lRjA5BpE1R_A2-dk9gEaHtt6Yfn8HJIa3FM1FnDaPHUAsEUuQ$)). If published, this will include your full peer review and any attached files.


**Do you want your identity to be public for this peer review?** If you choose “no”, your identity will remain anonymous but your review may still be made public.


For information about this choice, including consent withdrawal, please see our [Privacy Policy](https://urldefense.com/v3/__https:/track.editorialmanager.com/CL0/https:*2F*2Fwww.plos.org*2Fprivacy-policy/1/010f0195712220a5-16e0e99c-7812-4672-8018-ec0e82bb5c49-000000/Znly9Ya-HUKaG-3hUx2tJfWuPs5T8KU1xjHCxNPJUFs=201__;JSUl!!K-Hz7m0Vt54!jnTdFgA-EdV2Y3Czk5UKTDjpGzK0ppNCT4TAok2652lRjA5BpE1R_A2-dk9gEaHtt6Yfn8HJIa3FM1FnDaPHjTGosBo$).

Reviewer #1: No

Reviewer #2: No

[NOTE: If reviewer comments were submitted as an attachment file, they will be attached to this email and accessible via the submission site. Please log into your account, locate the manuscript record, and check for the action link "View Attachments". If this link does not appear, there are no attachment files.]

While revising your submission, please upload your figure files to the Preflight Analysis and Conversion Engine (PACE) digital diagnostic tool, [https://pacev2.apexcovantage.com/](https://urldefense.com/v3/__https:/pacev2.apexcovantage.com/__;!!K-Hz7m0Vt54!jnTdFgA-EdV2Y3Czk5UKTDjpGzK0ppNCT4TAok2652lRjA5BpE1R_A2-dk9gEaHtt6Yfn8HJIa3FM1FnDaPHfgFub2w$). PACE helps ensure that figures meet PLOS requirements. To use PACE, you must first register as a user. Registration is free. Then, login and navigate to the UPLOAD tab, where you will find detailed instructions on how to use the tool. If you encounter any issues or have any questions when using PACE, please email PLOS at [figures@plos.org](mailto:figures@plos.org). Please note that Supporting Information files do not need this step.
